# Modular Proteoglycan Perlecan/*HSPG2*: Mutations, Phenotypes, and Functions

**DOI:** 10.3390/genes9110556

**Published:** 2018-11-16

**Authors:** Jerahme R. Martinez, Akash Dhawan, Mary C. Farach-Carson

**Affiliations:** 1 Department of Mechanical Engineering, University of Delaware, Newark, DE 19716, USA; jerahme@udel.edu; 2 Department of Bioengineering, Rice University, Houston, TX 77005, USA; asd3@rice.edu; 3 Department of Diagnostic and Biomedical Sciences, University of Texas Health Science Center at Houston, School of Dentistry, Houston, TX 77054, USA

**Keywords:** perlecan, Schwartz-Jampel syndrome, dyssegmental dysplasia Silverman-Handmaker type

## Abstract

Heparan sulfate proteoglycan 2 (*HSPG2*) is an essential, highly conserved gene whose expression influences many developmental processes including the formation of the heart and brain. The gene is widely expressed throughout the musculoskeletal system including cartilage, bone marrow and skeletal muscle. The *HSPG2* gene product, perlecan is a multifunctional proteoglycan that preserves the integrity of extracellular matrices, patrols tissue borders, and controls various signaling pathways affecting cellular phenotype. Given *HSPG2*’s expression pattern and its role in so many fundamental processes, it is not surprising that relatively few gene mutations have been identified in viable organisms. Mutations to the perlecan gene are rare, with effects ranging from a relatively mild condition to a more severe and perinatally lethal form. This review will summarize the important studies characterizing mutations and variants of *HSPG2* and discuss how these genomic modifications affect expression, function and phenotype. Additionally, this review will describe the clinical findings of reported *HSPG2* mutations and their observed phenotypes. Finally, the evolutionary aspects that link gene integrity to function are discussed, including key findings from both in vivo animal studies and in vitro systems. We also hope to facilitate discussion about perlecan/*HSPG2* and its role in normal physiology, to explain how mutation can lead to pathology, and to point out how this information can suggest pathways for future mechanistic studies.

## 1. Introduction

The human gene heparan sulfate proteoglycan 2/*HSPG2* encodes for the secreted molecule perlecan, which is deposited in all basement membranes including those underlying epithelial and endothelial cells. It is also found in matrices produced in muscle, cartilage, and bone marrow. It plays a vital role in the formation of cardiovascular, neural and cartilaginous tissues. In humans, variations to the *HSPG2* gene produce severe developmental defects. Complete loss-of-function mutations are embryonically lethal, making it difficult to establish a complete genotype-phenotype relationship. To date, a number of *HSPG2* gene variants have been discovered and linked to a functional outcome and a clinical phenotype [1,2,3,4,5,6,7,8]. Although rare, mutations in *HSPG2* are associated with two classes of human skeletal disorders, known as Schwartz-Jampel syndrome (SJS; OMIM #255800) and dyssegmental dysplasia, Silverman-Handmaker type (DDSH; OMIM #224410). These two disorders are characterized by widespread developmental defects of all musculoskeletal tissues. The degree of severity in these two disorders depends on the location of the mutations and the extent of the protein core that is preserved, which determine the amount of functional protein that is produced and secreted into the various extracellular matrices (ECMs). Studies with the perlecan hypomorphic mouse [9] showed that when one allele of the perlecan gene was disrupted, an SJS phenotype was observed, with the amount of perlecan in fibroblasts, skeletal muscle, heart and kidney reduced by >90%. In addition to its role in development, perlecan also has been implicated in various pathologies associated with ECM remodeling and tissue repair, including that in cancer, diabetes, cardiovascular disease, and Alzheimer’s disease [10,11,12,13]. This review will describe existing views regarding *HSPG2* variations and mutations, and their impact on gene expression, protein function, and tissue phenotype.

## 2. *HSPG2*/Perlecan: Gene and Proteoglycan

**Figure 1:**
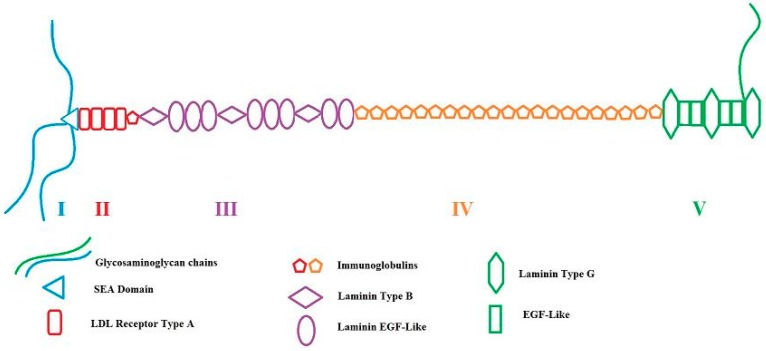
Representation of human heparan sulfate proteoglycan 2 (HSPG2)/perlecan proteoglycan with labelled domains I–V and subdomains. Three 3–4 glycosaminoglycan (GAG) chains are shown in this image where they are commonly attached on domain I. Additionally, a fourth chain appears variously on domain V. Adapted from Farach-Carson and Carson [29]. This article also contains a description of molecules known to be interacting with each domain.

The complex *HSPG2* gene encoding the proteoglycan perlecan is located on chromosome 1p36.1-p35 and spans over 120kbp of genomic DNA [14,15,16,17]. In humans, a total of 97 exons encode a core protein product that contains 4,391 amino acids and has a molecular weight of 467 kDa [1,18,19]. The addition of 3–4 glycosaminoglycan (GAG) side chains can extend the molecule over 750 kDa in size [20,21] making perlecan one of the largest monomeric matrix molecules. The core protein of perlecan consists of five domains, referred to as domains I–V (Figure 1), which are composed of tandemly repeating modular motifs arranged in a long linear fashion to resemble a “pearls-on-a-string” appearance that inspired the naming of the proteoglycan “perlecan” [22]. Domain I contains a Sperm, Enterokinase and Agrin (SEA) fold, which is found in other matrix and cell surface proteins [23,24]. To date no specific function of this fold has been demonstrated but it is known to appear in protein regions that are highly glycosylated. Domain I contains three GAG attachment sites that precede the SEA fold, which can be modified with either heparan sulfate (HS) or chondroitin sulfate (CS) chains depending on the cell or tissue source [24]. Heparin binding growth factors (HBGFs) involved in key development processes and wound healing bind to the GAG side chains of perlecan. During wound healing, perlecan’s GAG chains are cleaved by glycosaminoglycanases (GAGases), releasing HBGFs directly at the site of injury [25]. GAGases involved in HBGF release include heparanase [26] and sulfatases such as Sulfs 1 and 2 [27]. Domain II contains four low density lipoprotein (LDL) receptor motifs and one isolated immunoglobulin-like (IG) fold [18]. Both structures are held together with disulfide bonds and contribute to the compact modular shape of perlecan [28]. It is likely that this domain is involved in LDL and calcium signaling. It is also speculated that domain II is involved in wingless (Wnt) signaling, which is important for many developmental processes [29]. Perlecan’s modulation of Wnt has been demonstrated in various vertebrates [30]. Domain III contains both laminin epidermal growth factor (Egf) laminin IV type A (laminin B) domains [31]. Domain III forms an inflexible rod-like structure that is maintained by disulfide linkages in the laminin EGF domains. Fgf-18, found in developing growth plates and possesses mitogenic activity on chondrocytes, binds directly to domain III of perlecan [32]. In humans, domain IV is made up of 21 repeating Ig C2-type modules that are tandemly linked together. In general, Ig motifs are involved in adhesion, and found in many ECM and cell surface proteins. Domain IV of perlecan interacts with many ECM components including nidogen and fibronectin [33]. Proteins containing Ig repeats have been linked to provision of mechanical stability to flexible proteins that are large and modular, and typically found in tissues such as cartilage and muscle [34]. Only recently has perlecan been examined in this context [35]. Domain V contains three laminin G, four EGF motifs and has the fourth variable GAG attachment site. Domain V contains a region that when produced in a soluble form is known as endorepellin [36]. When the core protein is intact, perlecan possesses proangiogenic properties, while endorepellin has antiangiogenic activity [37]. Endorepellin has largely been studied for its role in controlling tumor angiogenesis and in neuroprotection following ischemic stroke [38,39]. Perlecan shares homology with a number of different proteins, owing to its unique ability to interact with so many partners and participate in a vast array of signaling pathways. The activity of perlecan depends on the cellular context and the form in which perlecan is presented. Post-translational variations that impact functions include GAG composition (HS versus CS content), proteolytic processing, exemplified by the matrix metalloproteinase-7 cleavage of perlecan that alters the semaphorin pathway and cell clustering [40], and GAGase trimming (by heparanase, chondroitinases, or the Sulfs). Interestingly, perlecan is one of few proteoglycans that can be substituted with three different types of GAGs, i.e., heparan, chondroitin, or keratan sulfate [41]. Additionally, various factors (i.e., cell source, aging, or injury) can influence GAG composition and therefore impact the function of perlecan [42,43,44]. Knox et. al. demonstrated that perlecans obtained from different cell sources differed in their ability to bind fibroblast growth factor (FGF) 2 and activate its receptors, despite all the tested perlecans having similar HS chains [44]. This study showed that even subtle changes to GAG structure can have profound effects on function.

## 3. Conservation of *HSPG2*

**Figure 2:**
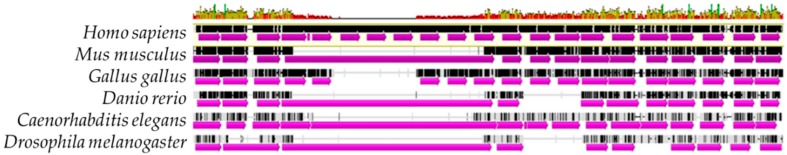
Conservation of *HSPG2* among common animal models. The perlecan domain IV cDNA sequence was analyzed in *Homo sapiens* (human), *Mus musculus* (mouse), *Gallus gallus* (chicken), *Danio rerio* (zebra fish), *Caenorhabditis elegans* (nematode), and *Drosophila melanogaster* (fruit fly). The alignment was performed using the software Geneious v5.4 [48]. The top graph represents consensus sequence identity among the species examined, with green and red representing high and low base pair conservation amongst all the listed organisms. All sequences are compared to *HSPG2* of *Homo sapiens*, which is highlighted in yellow at the top of the list. Sequences are shown in a grey color scale, representing low (white) to high (black) similarity to that of the reference cDNA sequence. The purple arrows parallel the sequence of individual Ig modules.

To illustrate the variation among the perlecan gene in various species, Figure 2 provides a cDNA sequence alignment of *HSPG2* with orthologs from commonly studied animal models. We focused on domain IV for our comparative sequence analysis because this region differs considerably among various species. Until recently, this domain was among the relatively least studied of the five domains, despite making up half the molecule. The differences in the number of Ig repeats across species is likely due to insertion or duplication events [45,46]. Although mouse perlecan lacks 7 Ig motifs found in humans, the protein identity amongst mammals remains nearly 90% [45], but is much lower in animals from other classes, such as *Danio rerio* having a less than 20% identity. In humans, a C-terminal region of domain IV (domain IV-3) has been shown to play a key role in cellular decision making, particularly in inducing cell cohesion such as occurs in formation of endothelia upon a basement membrane or condensation in early chondrogenesis [40,47].

Here, we will highlight some of the other major differences between the human form of perlecan and its orthologs. The mouse form of perlecan contains a unique integrin-mediated RGD binding site within its domain III, which is absent in human perlecan [49]. *Drosophila* and *C. elegans* do not have a domain I. The evolutionary origin of the perlecan gene was studied recently in hope of understanding the differences among species and to gain insight into its role in establishing tissue layers [45]. In this recent study, the perlecan gene was identified in the genome of early metazoans considered to have relatively simple tissue structures. In silico work revealed the perlecan gene was conserved in the genomes of *Trichoplax adhaerens*, a member of the Placozoan phylum defined by its small flattened amoeba-like body, and *Nematostella vectensis*, of the Cnidarian phylum that contains jellyfish and coral, when distinct tissue layers appear [45,50,51]. The perlecan gene was not found in the genomes of more ancient organisms such as the free-living unicellular Choanoflagellates or the aquatic and radial symmetric Ctenophores (e.g., comb jellies) [52,53]. Perlecan is also absent in *Capaspora owczarzaki*, a unicellular organism that garnered interest from the scientific community because it contains genes that closely relate to those of multicellular animals [54]. *N. vectensis* and *T. adhaerens* are considered morphologically simple animals and have been used to study tissue regeneration and evolution of multicellularity, respectively [55,56]. The perlecan gene in *T. adhaerens* is encoded by two separate, but adjacent genes, which house all five domains of the human gene in order. In *N. vectensis*, the perlecan gene was detected in cells forming key tissue boundaries and was activated during wound healing and the formation of new basement membranes [48]. Given perlecan’s complex gene structure, it is surprising this molecule has remained conserved over millions of years of evolution. This suggests an ancient key function, likely evolving with the emergence of tissue layers and the need for repair of such tissues after wounding [48].

## 4. *HSPG2* Variants and Homologues

A few *HSPG2* splice variants, unrelated to a human condition, have been identified in various human tissue sources and cell lines. Although, the consequence and/or biological function of these alternative forms still need to be elucidated [57]. The first *HSPG2* splice variant, referred to as “miniperl”, was discovered by Dodge et al. (published in the NCBI database under the Accession number AF479675) in the human colon carcinoma cell line WiDr/HT29. This shortened form arises either from alternate splicing or use of alternative transcriptional start/stop sites events occurring in domain I. In the human mast cell line, HMC-1, domain I and domain V fragments were identified and examined in in vitro assays, the latter of which includes endorepellin sequences and retains anti-angiogenic activity [58]. An examination of all reported *HSPG2* transcript variants, reveals products corresponding to either the C-terminal or N-terminal domains, which may play separate roles in wound healing and tissue regeneration, although these products have yet to be characterized [57].

The two most commonly studied *HSPG2* homologues are those found in *C. elegans* and Drosophila. The *C. elegans*, the *HSPG2* homologue is *Unc-52* [59]. *Unc* mutants were first discovered for their role within the body-wall of muscle cells [60]. *Unc-52* mutated phenotype is characterized by retarded sarcomere construction and progressive paralysis, hence the name “*Unc*” for uncoordinated movement [61]. Unlike human *HSPG2*, *Unc-52* codes for several functional isoforms of perlecan. In general, longer isoforms are involved in the attachment of the myofilament lattice to the muscle cell membrane, while the role of shorter isoforms is less clear. Unc-52 contributes to gonadogenesis through regulation of growth factor signaling and by providing structural support to the tissue surrounding the gonad [62]. The Unc-52 depleted gonad basement membrane interferes with gonadal cell adhesion signaling and affects the migration of gonadal leader cells [62,63]. *Unc-52* mutations are associated with ECM remodeling defects in developing organs [64]. *Unc-52* splicing events are regulated *Smu*-1 and *Smu*-2 (suppressor of *Mec* and *Unc* defects) [65] *Mec-8* (mechanosensory abnormality) [66], *Hrp-2* (human HnRNP A1 homolog) [67], and *Ccar-1* (cell division cycle and apoptosis regulator 1 homolog) [68].

Trol, for terribly reduced optic lobes, is the *Drosophila* homologue of the vertebrate protein perlecan, [69]. Trol regulates critical development signaling pathways, such as Wnt and Indian hedgehog, and coordinates cell movement to establish cell and tissue layers. Trol is highly expressed in the developing central nervous system and the periphery, especially at the motor axon trajectories [70]. Hence its name, Trol modulates semaphorin-mediated repulsive axon guidance in the optic lobes. It has been shown Trol can augment semaphorin suppression of focal adhesion kinase activation, suggesting Trol supports the antagonistic effect of semaphorin on integrin signaling [70]. Interestingly, this same mechanism of focal adhesion kinase (FAK) suppression was found to be conserved in human cells undergoing cohesive events [40,47]. Similarly, during gastrulation Trol directs the movement of mesoderm cells to form a single mesoderm cell layer underlying the ectoderm. This process requires cooperation of FGF signaling. Mutations to the *Trol* gene not only modulate key signaling processes, but also influence cell behavior through rearrangements of the territorial matrix. Such is the case seen during *Drosophila* hematopoiesis, where perlecan is expressed along the thin basement membrane surrounding the blood progenitors. Here, perlecan regulates blood cell differentiation, through modulation of Hedgehog signaling, and provides structural support of the ECM of the lymph gland [71]. The Wnt signaling pathway is also regulated by Trol and is important for formation of pre and postsynaptic structures of the neuromuscular junction [30].

## 5. Developmental Expression of Perlecan

Perlecan expression and deposition begin in the early stages of embryogenesis, were it has been well-characterized in murine models. Perlecan was detected along the cell surface of blastomeres at the two-cell stage and during the attachment phase of implantation at the exterior surface of the trophectoderm [72,73]. Following implantation, perlecan accumulates throughout the developing cardiovascular system and at sites of cartilage primordia [74,75]. At embryonic day 10.5 (E10.5), perlecan is found in vascularized tissues such as the heart, pericardium, blood vessels walls, and in cartilage primordia [74,75]. The highest deposition of perlecan occurs in cartilage undergoing endochondral ossification, such as the primordium of vertebral bodies and rib cartilage. At later stages of development, perlecan is expressed throughout the basal lamina of the embryo and organs such as the lung, kidney, liver, gastrointestinal tract and brain [75].

## 6. *HSPG2* Associated Skeletal Defects

A number of mutations have been identified in both SJS and DDSH), but the exact genotype-phenotype correlation for each of these mutations has not been proven experimentally [5,6,7,76]. As mentioned above, the degree of severity inversely correlates with the amount of perlecan being deposited into the ECM [77]. The SJS phenotype is the relatively milder of the two conditions characterized by myotonia and chondrodysplasia [78,79]. Perlecan mutations associated with SJS lead to reduced levels of normal perlecan secretion into the ECM [6]. Patients with SJS survive but experience widespread skeletal abnormalities including reduced stature, facial dimorphism, pigeon breast, and shortened long bones. The neonatal lethal condition DDSH is caused by functional null mutations to *HSPG2*, which completely prevent perlecan secretion into the ECM [2]. Clinical features of DDSH include dwarfism, short and bowed limbs, flat facial features, anisospondyly, and encephalocele [80].

## 7. Mechanisms Underlying Perlecan Developmental Defects

The mechanism underlying perlecan disorders have been studied in mice. Perlecan-null mice, mimicking the DDSH phenotype, display normal formation of basement membranes during the first few days of development, but these soon deteriorate at areas undergoing increased mechanical stress such as contraction of the myocardium and expansion of brain vesicles [81,82,83]. Perlecan-null mice typically die around embryonic day (E) 10–12, attributed to heart and brain defects. In the heart, the loss of perlecan weakens the basements membranes around the heart wall, leading to a “leaky” interface between cardiomyocytes and surrounding endothelial cells [81,83]. As a result, embryos die of heart arrest from blood leaking into the pericardial cavity [81]. The cardiomyocytes maintain proper sarcomere form, tight junctions and have functional expression of ion channels, suggesting defects are related to loss of basement membrane integrity [81,83].

Cephalic defects on the surface appear to be quite strange given that perlecan is not expressed in the central nervous system [75]. Exencephaly usually occurs from improper neuronal tube closure, however, perlecan-null mice have a normal neural tube and proper closure of the neuropores [81,82]. Under normal conditions there is a solid layer of ectodermal cells encompassing the brain tissue, but in the absence of perlecan, small clefts about 20–30 µm wide are formed. Cephalic mesenchyme moves through these clefts and invade the ectoderm layer. The barrier separating the brain tissue from ectoderm is disrupted allowing the brain to fuse with the neighboring ectoderm. The laminar architecture surrounding the brain is severely distorted indicating the basement membrane barrier is lost [81]. The embryos also develop holes in their fore- and midbrain and have collapsed brain vesicles. Perlecan-null mice also experience severe bleeding within several tissues, such as the lung, skin and brain, caused by weakening of the blood vessel wall.

Reduced levels of perlecan in mice, mimicking the SJS phenotype, lead to failure of the chondro-osseous junction of developing bones [82]. The reduction of perlecan interferes with normal growth plate organization, causing bones to become shorter, thicker and misshapen. This is commonly seen in long bones, sternum, and innominate bone. Under normal circumstances, chondrocytes form columnar structures of cell layers (i.e., resting, proliferative, pre-hypertrophic and hypertrophic zones), but when perlecan expression is reduced these zones become highly disorganized and expand [81,82]. The chondro-osseous junction is lost and chondrocytes from the perichondrium layer invade the surrounding tissue and generate ectopic ossification [81]. The matrix of the perichondrium layer otherwise maintains normal levels of glycosaminoglycans and aggrecan, and maintains proper organization of collagen and fibronectin fibrils, yet overall tissue architecture is lost [76]. The catabolic turnover of perlecan’s HS chains by GAGases, such as heparanase and chondroitinase, at the chondro-osseous junction supports vascular endothelial growth factor (VEGF) signaling and promotes angiogenesis for cartilage matrix remodeling and bone formation [84,85]. At the hypertrophic zone of the growth plate, intact perlecan containing GAGs chains acts as barrier separating the mineralized bone tissue from cartilage. Perlecan deficient mice develop brittle bone due to changes in bone elastic modulus, mineral density, and cortical bone thickness [86]. Poor bone quality in perlecan deficient mice results from the disruption of the lacunar-canilicular system (LCS) of mineralized bone, where perlecan is part of the pericellular matrix (PCM) surrounding the osteocytic processes that preserve fluid flow throughout bone tissue [87]. When perlecan levels are reduced, there is encroachment of the canalicular wall and decreased pericellular space, yet another barrier function of perlecan, as it prevents mineral formation. This in turn affects solute transport through the LCS. Disrupting the normal fluid flow pattern impacts the amount of drag force and shear stress experienced by osteocytes, and thus forces bone to adapt differently [88]. Perlecan is thought to function as an osteocyte sensing tether, transmitting extracellular fluid flow drag forces to the osteocyte cell surface [86,87,88]. The abnormal bone loading response observed in perlecan deficient mice best supports this claim. Increasing evidence suggests perlecan contributes to the mechanical stability of tensional and weight bearing tissues. Perlecan is part of the chondrocyte PCM in cartilage, where it interacts with collagen type VI to provide structural support, protection from compressive loading, and facilitate chondrocyte mechanotransduction [89,90,91,92]. Similarly, perlecan is involved in maintaining muscle composition and mass under loaded/unloaded conditions [93,94].

**Figure 3:**
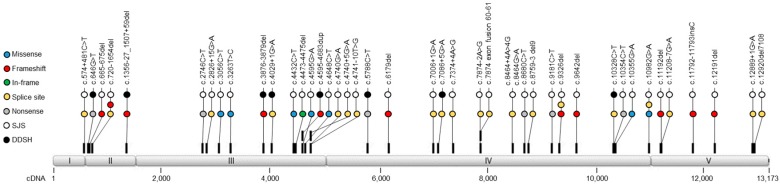
Location of *HSPG2* mutations identified in Schwartz-Jampel syndrome (SJS) and dyssegmental dysplasia, Silverman-Handmaker type (DSSH) patients. Schematic representation of *HSPG2* domain (I–V) encoding organization with corresponding locations of mutations. Mutation types are indicated by colored circles, with white and black indicating whether the mutation was identified in either a SJS or a DDSH patient, respectively. Mutations are identified by their cDNA sequence (Genbank M85289.1) as depicted below and follow the accepted nomenclature system [96].

Mutations nullifying the perlecan gene (Figure 3) are rare and lethal, making it difficult to study the mechanism underlying such mutations and their effects. Although there have been a number of reported cases of DDSH, only eight cases have been molecularly characterized. Here, we will discuss those few mutations associated with the complete loss of perlecan secretion and the resulting clinical phenotype of DDSH. Figure 3 is used to reference the position of subsequent mutations to be discussed. Although all known mutations associated with perlecan disorders are shown (including those associated with SJS), not all will be discussed in this review. In addition, a single mutation alone does not represent a patient phenotype; rather a clinical outcome is observed when both alleles are affected as in a compound heterozygous or homozygous mutation. The first study to uncover *HSPG2* null mutations was carried out by Arikawa-Hirasawa and colleagues [2]. This study identified perlecan null mutations in three patients (cases 1–3), including a pair of siblings born to consanguineous parents and a third unrelated patient. The two siblings were homozygous for an 89 base pair duplication between base pairs 4683 and 4684 of exon 34 of *HSPG2*, corresponding to domain III. The other unrelated patient was heterozygous for point mutations at the 5′ donor site of intron 52 and within exon 73 (domain IV), leading to the skipping of exons 52 and 73, respectively. Shortened *HSPG2* transcription products were observed in all three patients, likely caused by a frameshift that introduced a premature stop codon. Immunological analysis demonstrated that the truncated perlecan core protein was not detected in the PCM of patient derived cartilage tissue; instead, the mutant fragments were retained intracellularly where they could be subjected to proteolytic degradation. Reiubland and colleagues [7] identified a fourth patient (case 4) having a homozygous four base pair deletion, c.3876–3879 delGTGC, in exon 31 of domain III. Similar to the other confirmed cases, this mutation created a frameshift and introduced a premature stop codon in exon 32. In another study, three patients (cases 5–7) from two different families were diagnosed with DDSH [95]. Two of these patients, born from nonconsanguineous parents and of the same family, were heterozygous for nonsense mutations c.646 G > T (exon 7) and c.5788C > T (exon 46). The third patient from this study, not related to the previous two and with consanguineous parents, had a homozygous deletion at exon 12 (c.1356-27_1507 + 59del). In this case, the parents were both heterozygous suggesting an autosomal recessive condition. Recently, an eighth individual (case 8) was reported in only one of twins born in a dizygotic pregnancy. The affected twin brother was homozygous for a c.4029 + 1 G > A mutation within exon 32, while the female twin sister was healthy having normal fetal anatomy and growth. The pregnancy was from consanguineous parents, for which the mother was a heterozygous carrier and state of the father remains unknown. Although a limited amount of human patient data exists, there are some clear observations from which we can gain insight. For instance, DDSH appears to result only when both alleles are mutated. As to date, a DDSH patient having only a single heterozygous mutation has yet to be identified. Additionally, mutations at the C-terminal end may be more catastrophic, in that they create shortened non-functional premature forms or disrupt key domains involved in protein folding.

However, not all truncations completely prevent perlecan secretion and loss of function. It appears that terminations occurring at the C-terminal region of the protein core are better tolerated, and the result is the less severe SJS rather than DDSH. For example, a SJS patient was diagnosed having a heterozygous transition mutation at the last nucleotide of exon 64 in allele 1 (c.8464 G > A) and had a 9 base pair deletion at the acceptor junction of intron 66 and exon 67 in allele 2 (c.8759-3del9) [6]. These mutations resulted in the skipping of exons 64 and created abnormal splicing with retention of intron 66 or skipping of exon 67, respectively. Interestingly, the c.8464 position appears to be a hot spot for mutations, as it has appeared in multiple SJS patients [1,5,6]. Similarly, another SJS patient was found to have truncations that exclusively removed large regions from the C-terminal region of domain V [6]. In this case the patient was diagnosed having a homozygous deletion that removed 7108 base pairs beginning at the 5′ region of exon 96 up to the 3′ flanking sequence of the *HSGP2* gene (12920del7108), which lead to the retention of intro 95 or the failure to splice introns 94 and 95. As a result, the two transcript products created protein fragments lacking ~35 and ~64 amino acids from the C-terminus of domain V, respectively.

The SJS patients lacking domain V tend to show skeletal defects much earlier than those patients that can produce a mutant full-length version of perlecan. This suggests domain V plays a key role in the early onset of chondrodysplasias. Normal domain V function primarily involves cell-matrix interactions; it has shown both pro- and anti-angiogenic activity through endothelial cell integrin binding [36,97]. Furthermore, it has been shown that the C-terminal domain V contributes to supramolecular assembly and cell-basement membrane connections through β1-integrin cell adhesion, heparin, nidogen and fibulin-2 binding [98]. Thus, it is possible that domain V binds to chondrocyte integrin receptors and stabilizes the cartilage matrix, contributing to normal cartilage development [6]. Therefore, with the loss of domain V, the cartilage matrix does not develop properly, leading to the chondrodysplasia symptoms associated with SJS.

Two potential mechanisms have been proposed for the myotonia symptoms associated with the SJS caused by perlecan mutation. First, full length perlecan may serve to cluster acetylcholinesterase at the membrane of the neuromuscular junction by binding to the enzyme at HS binding domains and the membrane dystroglycan at domain V [46,99]. The loss of localization function may contribute to a slower degradation of acetylcholine that results in the hyperexcitability exhibited in SJS. This loss of localization function may occur through mutations in domain V that prevent the protein from binding to the membrane. Second, domain V may bind directly to sodium or chlorine ion channels on the muscle, modifying their function and leading to the indicated hyperexcitability myotonia symptoms of SJS [1]. Domain V also has been associated with conditions beyond skeletal defects. Specifically, an adenine to guanine substitution (c.11827 G > A; NM_005529.6) results in an alanine to threonine amino acid replacement (A3943T) that appears in certain SJS patients suffering from ectopic mineralization in the kidney resulting in juvenile nephrolithiasis and associated osteopenia (Lada Beara Lasic and Farach-Carson, unpublished). A proposed mechanism for this development that is presently under investigation is that the mutation produces a new hydrogen bond, thus changing the protein folding pattern and revealing potential calcium binding sites that serve as nucleators for calcium deposition.

## 8. Conclusions

Perlecan is a modular multifunctional ECM proteoglycan that serves vital roles in development, wound healing and tissue morphogenesis. It influences multiple signaling pathways that determine key cell fate decisions and tissue phenotype. It is ancient and highly conserved across species, indicating that selective pressures during evolution have favored retention of the gene encoding the protein as a long modular monomer. Given its essential role in tissue formation during development, mutations in the *HSPG2* gene are particularly difficult to study since most are embryonic or neonatal lethal. Those recorded non-lethal human mutations result in two skeletal defects: SJS and DDSH, and perhaps in ectopic mineralization. *HSPG2* mutations in viable organisms typically occur near the region of the gene encoding the C-terminus, allowing most of the protein to be secreted. In mouse models, perlecan-null mice suffered lethal complications in the heart and brain in addition to hemorrhage in various other tissues and organs. In hypomorphic mice with reduced levels of perlecan, the barrier functions of the protein were not sufficiently met, leading to complications in bone development and integrity. In humans, four cases of DDSH have been characterized. Milder mutations that occur closer to the C-terminus in domains IV and V have shown to produce the milder SJS phenotype. Much work remains to be done toward the investigation of non-lethal perlecan mutations and variations that may more subtly influence phenotype. The growing access to whole genome sequencing in large patient populations and popular personal sequence repositories such as 23andMe, Inc. (Mountain View, CA, USA) and AncestryDNA^®^ (Ancestry LLC, Lehi, UT, USA) may provide the opportunity to explore further the mutational and variant landscape for this fascinating gene.
